# *N*-*tert*-But­oxy­carbonyl-α-(2-fluoro­benzyl)-l-proline

**DOI:** 10.1107/S1600536813019788

**Published:** 2013-07-24

**Authors:** P. Rajalakshmi, N. Srinivasan, R. V. Krishnakumar, Ibrahim Abdul Razak, Mohd Mustaqim Rosli

**Affiliations:** a Department of Physics, Thiagarajar College, Madurai 625 009, India; b X-ray Crystallography Unit, School of Physics, Universiti Sains Malaysia, 11800-USM, Penang, Malaysia

## Abstract

In the title compound, C_17_H_22_FNO_4_, the pyrrolidine ring adopts an envelope conformation with the disordered com­ponents of the methylene C atom, with site occupancies of 0.896 (7) and 0.104 (7), being the flap on either side of the mean plane involving the other atoms of the ring. The carb­oxy­lic acid group forms dihedral angles of 72.06 (11) and 45.44 (5)° with the *N*-*tert*-but­oxy­carbonyl group and the 2-fluoro­benzyl group, respectively. In the crystal, two-dimensional layers of mol­ecules parallel to (001) are built through an *R*_4_^4^(23) motif generated *via* O—H⋯O, C—H⋯O and C—H⋯F inter­actions, and an *R*_2_^2^(11) motif generated by C—H⋯O and C—H⋯F inter­actions.

## Related literature

For general background, see: Taylor *et al.* (1998 ▶); Jeng *et al.* (2002 ▶); Anderson *et al.* (2004 ▶); Ryder *et al.* (2000 ▶). For biological activity of the title compound, see: Tamazyan *et al.* (2004 ▶). For graph-set notation of hydrogen bonding, see: Bernstein *et al.* (1995 ▶). For puckering parameters, see: Cremer & Pople (1975 ▶).
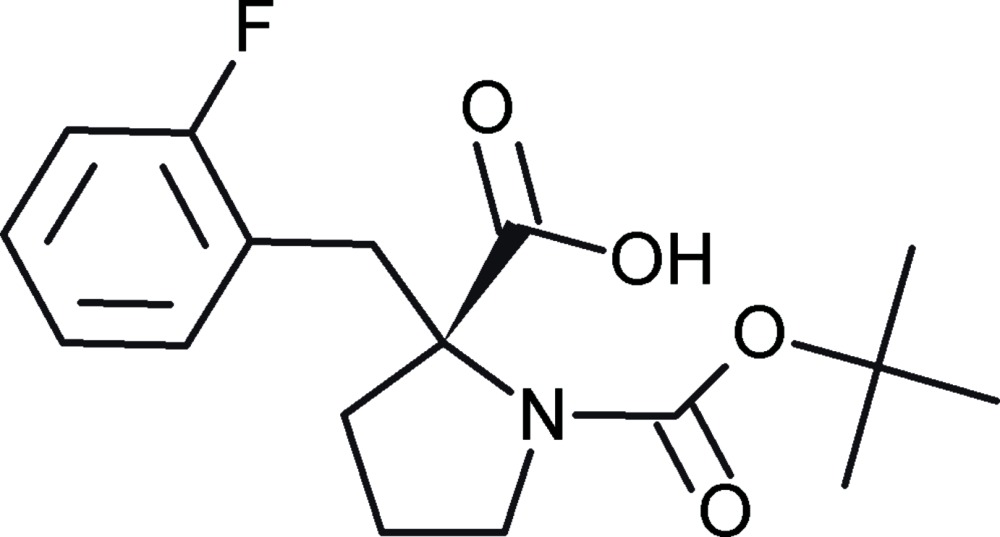


## Experimental

### 

#### Crystal data

C_17_H_22_FNO_4_
*M* *_r_* = 323.36
Orthorhombic, 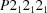

*a* = 10.4777 (1) Å
*b* = 12.4283 (2) Å
*c* = 13.1550 (2) Å
*V* = 1713.04 (4) Å^3^
*Z* = 4
Mo *K*α radiation
μ = 0.10 mm^−1^
*T* = 100 K
0.54 × 0.34 × 0.24 mm

#### Data collection

Bruker Kappa APEXII diffractometer
Absorption correction: multi-scan (*SADABS*; Sheldrick, 2008 ▶) *T*_min_ = 0.962, *T*_max_ = 0.977
14001 measured reflections
3426 independent reflections
2712 reflections with *I* > 2σ(*I*)
*R*_int_ = 0.031
Standard reflections: 0

#### Refinement

*R*[*F* ^2^ > 2σ(*F* ^2^)] = 0.050
*wR*(*F* ^2^) = 0.114
*S* = 1.06
3426 reflections
225 parameters
8 restraints
H atoms treated by a mixture of independent and constrained refinement
Δρ_max_ = 0.37 e Å^−3^
Δρ_min_ = −0.32 e Å^−3^

### 

Data collection: *APEX2* (Bruker, 2009 ▶); cell refinement: *SAINT* (Bruker, 2009 ▶); data reduction: *SAINT*; program(s) used to solve structure: *SHELXS97* (Sheldrick, 2008 ▶); program(s) used to refine structure: *SHELXL97* (Sheldrick, 2008 ▶); molecular graphics: *PLUTON* (Spek, 2009 ▶); software used to prepare material for publication: *SHELXL97*.

## Supplementary Material

Crystal structure: contains datablock(s) I, global. DOI: 10.1107/S1600536813019788/gk2586sup1.cif

Structure factors: contains datablock(s) I. DOI: 10.1107/S1600536813019788/gk2586Isup2.hkl

Additional supplementary materials:  crystallographic information; 3D view; checkCIF report

## Figures and Tables

**Table 1:** Hydrogen-bond geometry (Å, °)

*D*—H⋯*A*	*D*—H	H⋯*A*	*D*⋯*A*	*D*—H⋯*A*
C15—H15*A*⋯F1^i^	0.95	2.59	3.378 (3)	141
C16—H16*A*⋯O1^i^	0.95	2.60	3.541 (3)	173
O1—H1⋯O3^ii^	0.89 (3)	1.73 (3)	2.611 (2)	173 (3)

Symmetry codes: (i) 
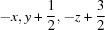
; (ii) 
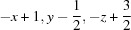
.

**Fig. 1.**
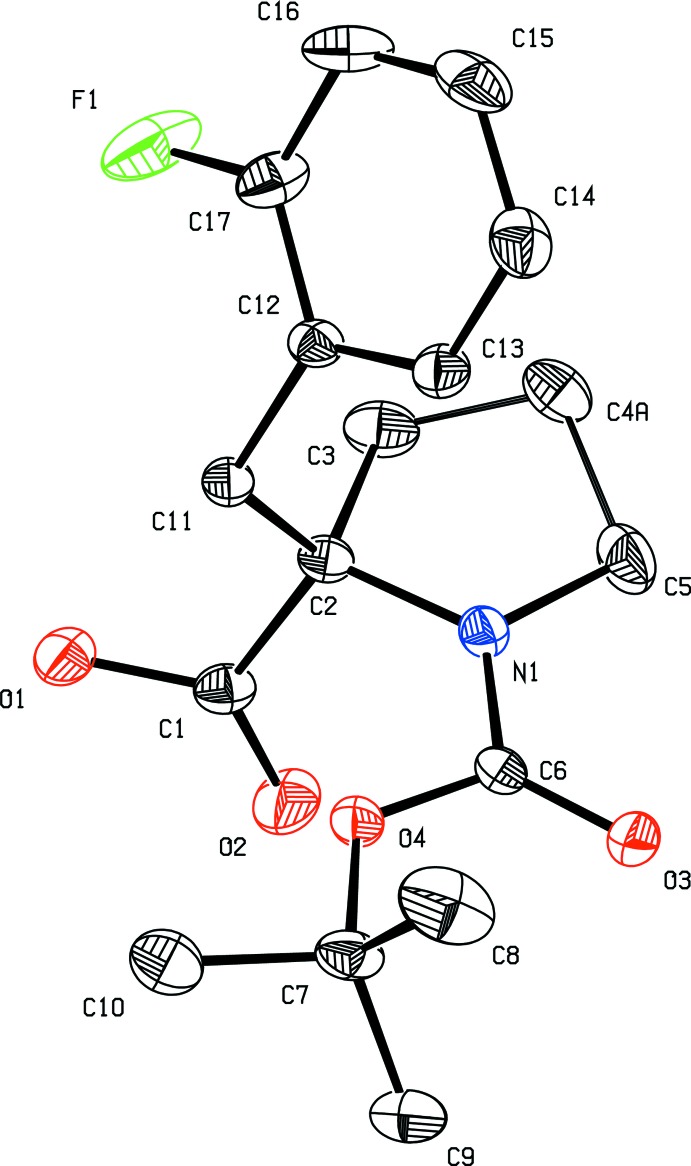
Molecular structure of the title compound showing the major component of the disorder. Displacement ellipsoids for non-H atoms are drawn at the 50% probability level.

**Fig. 2.**
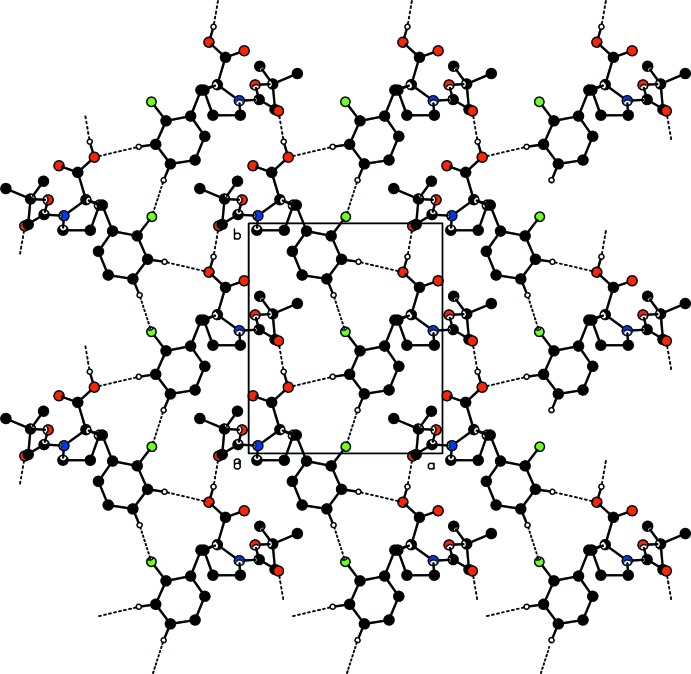
Part of the crystal structure of the title compound, showing R22(11) and R44(23) motifs through a combination of O—H···O, C—H···O and C—H···F hydrogen bonds and the formation of a two dimensional layer parallel to the ab plane. For the sake of clarity, H atoms not involved in hydrogen bonding have been omitted.

## supplementary crystallographic information

### Comment

Modified amino acids are known to enhance the chemical, physical and biological properties of proteins (Anderson *et al.*, 2004). Also, due to their structural diversity and functional versatility, they are widely used as chiral building blocks and molecular scaffolds in pharmaceutics (Taylor *et al.*, 1998; Ryder *et al.*, 2000; Jeng *et al.*, 2002). *N*-Butoxycarbonyl-(S)-α-benzyl proline, a closely related analogue of the title compound N-*tert-*butoxycarbonyl-α-(2-fluorobenzyl)-*L*-proline, is a potential non-nucleoside reverse transcriptase inhibitor in anti-human-immunodeficiency virus type-1 (Tamazyan *et al.*, 2004).

The present paper describes the crystal structure of the title compound (Fig. 1), which crystallizes in the orthorhombic space group *P*2_1_2_1_2_1_. It is a modified amino acid with the N-terminus protected by a *tert*-butyloxycarbonyl (Boc) group and the C^α^ (C2) H atom replaced by a 2-fluorobenzyl group. In the pyrrolidine ring, the C4 atom of the ring displays positional disorder with site-occupation factors of 0.896 (7) and 0.104 (7). The pyrrolidine ring (N1/C2/C3/C4A/C5) adopts the envelope conformation with the C4A atom deviating from the plane defined the remaining ring atoms by 0.5677 (4) Å. Puckering parameters calculated for this ring are of Q = 0.369 (3) Å, φ = 285.7 (3)° (Cremer & Pople, 1975). The dihedral angles between the mean plane of the carboxylic acid group, N-Boc and 2-methyl-2-fluorobenzene are 72.06 (11)° and 45.44 (5)°, respectively.

The molecules are linked by a combination of O—H···O, C—H···O and C—H···F hydrogen bonds. The carboxylic O1 acts as a donor to the carbonyl O3 at (*-x + 1, y - 1/2, -z + 3/2*) forming chains parallel to the *b* axis through *C*(7) motifs (Bernstein *et al.*, 1995). These 2_1_ screw-generated parallel chains are interconnected through C15—H15A···F1 (*-x, y + 1/2, -z + 3/2*) and C16—H16A···O1 (*-x, y + 1/2, -z + 3/2*) hydrogen bonds leading to a layer parallel to the *ab* plane. The characteristic building units of this layer are an *R*^4^_4_(23) ring generated by all the hydrogen-bonds and an *R*^2^_2_(11) generated exclusively by C—H···O and C—H···F hydrogen bonds (Fig. 2).

### Experimental

A mixture of 2-(2-fluorobenzyl)-*L*-proline (1.0 mmol) and tetramethylammonium hydroxide pentahydrate (1.2 mmol) in acetonitrile (10 ml) was stirred for 30 min. After 30 min, Boc_2_O (2.0 mmol) was added and stirred continuously for 2 d. The acetonitrile was removed *in vacuo* and residue was partitioned between ether (20 ml) and water (10 ml). The aqueous layer was washed with ether (10 ml) and acidified with 10% aqueous citric acid to pH 3–4. The aqueous layer was extracted with ethyl acetate (3 × 10 ml) and combined organic extracts were washed with brine solution (1 × 10 ml), dried over Na_2_SO_4_, and concentrated to yield *N*-*tert*-butoxycarbonyl-α-(2-fluorobenzyl)-*L*-proline (m.p. 430-433 K) as a white solid. Crystals were grown from ethanolic solution by slow evaporation at room temperature.

### Refinement

All H atoms, except hydroxy H1 atom, were placed at geometrically calculated positions (0.99 Å for methylene C—H, 0.98 Å for methyl C—H and 0.95 Å for aromatic C—H) and refined using a riding model. The U_iso_ values of all H atoms were constrained to 1.2U_eq_ (1.5 times for hydroxyl and methyl H atoms) of the respective atom to which the H atom bonds. The hydroxy H1 atom was freely refined. In the pyrrolidine ring, the C4 atom exhibits disorder (resolved into C4A and C4B) and the same was modelled using SIMU and SADI restraints leading to site-occupancies of 0.896 (7) and 0.104 (7). In the absence of significant anomalous scattering effects 1492 Friedel pairs were merged. The enantiomer has been assigned by reference to an unchanging chiral centre in the synthetic procedure.

### Crystal data

**Table:**

C_17_H_22_FNO_4_	*F*(000) = 688
*M**_r_* = 323.36	*D*_x_ = 1.254 Mg m^−^^3^
Orthorhombic, *P*2_1_2_1_2_1_	Mo *K*α radiation, λ = 0.71073 Å
Hall symbol: P 2ac 2ab	Cell parameters from 3426 reflections
*a* = 10.4777 (1) Å	θ = 2.3–30.0°
*b* = 12.4283 (2) Å	µ = 0.10 mm^−^^1^
*c* = 13.1550 (2) Å	*T* = 100 K
*V* = 1713.04 (4) Å^3^	Block, colourless
*Z* = 4	0.54 × 0.34 × 0.24 mm

### Data collection

**Table:**

Bruker Kappa APEXII diffractometer	3426 independent reflections
Radiation source: fine-focus sealed tube	2712 reflections with *I* > 2σ(*I*)
Graphite monochromator	*R*_int_ = 0.031
φ and ω scans	θ_max_ = 32.6°, θ_min_ = 2.3°
Absorption correction: multi-scan (*SADABS*; Sheldrick, 2008)	*h* = −13→15
*T*_min_ = 0.962, *T*_max_ = 0.977	*k* = −18→18
14001 measured reflections	*l* = −18→18

### Refinement

**Table:**

Refinement on *F*^2^	Primary atom site location: structure-invariant direct methods
Least-squares matrix: full	Secondary atom site location: difference Fourier map
*R*[*F*^2^ > 2σ(*F*^2^)] = 0.050	Hydrogen site location: inferred from neighbouring sites
*wR*(*F*^2^) = 0.114	H atoms treated by a mixture of independent and constrained refinement
*S* = 1.06	*w* = 1/[σ^2^(*F*_o_^2^) + (0.043*P*)^2^ + 0.5231*P*] where *P* = (*F*_o_^2^ + 2*F*_c_^2^)/3
3426 reflections	(Δ/σ)_max_ < 0.001
225 parameters	Δρ_max_ = 0.37 e Å^−^^3^
8 restraints	Δρ_min_ = −0.32 e Å^−^^3^

### Special details

**Table:**

Geometry. All esds (except the esd in the dihedral angle between two l.s. planes) are estimated using the full covariance matrix. The cell esds are taken into account individually in the estimation of esds in distances, angles and torsion angles; correlations between esds in cell parameters are only used when they are defined by crystal symmetry. An approximate (isotropic) treatment of cell esds is used for estimating esds involving l.s. planes.
Refinement. Refinement of *F*^2^ against ALL reflections. The weighted *R*-factor *wR* and goodness of fit *S* are based on *F*^2^, conventional *R*-factors *R* are based on *F*, with *F* set to zero for negative *F*^2^. The threshold expression of *F*^2^ > σ(*F*^2^) is used only for calculating *R*-factors(gt) *etc*. and is not relevant to the choice of reflections for refinement. *R*-factors based on *F*^2^ are statistically about twice as large as those based on *F*, and *R*-factors based on ALL data will be even larger.

### Fractional atomic coordinates and isotropic or equivalent isotropic displacement parameters (Å^2^)

**Table:**

	*x*	*y*	*z*	*U*_iso_*/*U*_eq_	Occ. (<1)
F1	−0.00102 (13)	0.97131 (13)	0.77647 (14)	0.0493 (4)	
O1	0.29559 (14)	0.71232 (12)	0.76893 (13)	0.0272 (3)	
H1	0.320 (3)	0.645 (3)	0.756 (3)	0.055 (9)*	
O2	0.47731 (16)	0.74954 (12)	0.68734 (14)	0.0346 (4)	
O3	0.65381 (14)	1.01134 (12)	0.77232 (14)	0.0296 (4)	
O4	0.53366 (13)	0.89798 (11)	0.86819 (11)	0.0227 (3)	
N1	0.45214 (15)	0.96664 (13)	0.72598 (13)	0.0191 (3)	
C1	0.38068 (19)	0.77841 (16)	0.72823 (16)	0.0205 (4)	
C2	0.33880 (18)	0.89677 (15)	0.73257 (15)	0.0177 (4)	
C3	0.2696 (2)	0.92068 (18)	0.63034 (17)	0.0272 (5)	
H3A	0.2918	0.8660	0.5786	0.033*	0.896 (7)
H3B	0.1759	0.9210	0.6398	0.033*	0.896 (7)
H3C	0.2326	0.8539	0.6017	0.033*	0.104 (7)
H3D	0.2001	0.9735	0.6407	0.033*	0.104 (7)
C4A	0.3162 (2)	1.0299 (2)	0.59876 (18)	0.0271 (7)	0.896 (7)
H4A	0.2680	1.0875	0.6337	0.032*	0.896 (7)
H4B	0.3084	1.0398	0.5244	0.032*	0.896 (7)
C4B	0.3694 (15)	0.9656 (18)	0.5602 (4)	0.058 (7)	0.104 (7)
H4C	0.3306	1.0131	0.5083	0.070*	0.104 (7)
H4D	0.4169	0.9072	0.5257	0.070*	0.104 (7)
C5	0.4573 (2)	1.02965 (19)	0.63191 (17)	0.0299 (5)	
H5A	0.5122	0.9947	0.5804	0.036*	0.896 (7)
H5B	0.4887	1.1036	0.6447	0.036*	0.896 (7)
H5C	0.5453	1.0334	0.6049	0.036*	0.104 (7)
H5D	0.4248	1.1036	0.6427	0.036*	0.104 (7)
C6	0.55438 (19)	0.96175 (15)	0.78805 (16)	0.0212 (4)	
C7	0.6244 (2)	0.89395 (17)	0.95460 (17)	0.0247 (4)	
C8	0.6362 (3)	1.0048 (2)	1.0019 (2)	0.0452 (7)	
H8A	0.6801	1.0531	0.9545	0.068*	
H8B	0.6852	0.9998	1.0651	0.068*	
H8C	0.5509	1.0332	1.0166	0.068*	
C9	0.7514 (2)	0.8487 (2)	0.9205 (2)	0.0371 (6)	
H9A	0.7944	0.9009	0.8764	0.056*	
H9B	0.7373	0.7817	0.8828	0.056*	
H9C	0.8048	0.8341	0.9801	0.056*	
C10	0.5574 (3)	0.8169 (2)	1.02650 (18)	0.0337 (5)	
H10A	0.5444	0.7476	0.9924	0.050*	
H10B	0.4745	0.8470	1.0462	0.050*	
H10C	0.6099	0.8063	1.0873	0.050*	
C11	0.25451 (19)	0.92051 (15)	0.82578 (15)	0.0186 (4)	
H11A	0.1825	0.8690	0.8263	0.022*	
H7B	0.3054	0.9077	0.8880	0.022*	
C12	0.20116 (18)	1.03351 (16)	0.82987 (14)	0.0182 (4)	
C13	0.2744 (2)	1.12161 (15)	0.85860 (16)	0.0212 (4)	
H13	0.3613	1.1109	0.8764	0.025*	
C14	0.2240 (2)	1.22470 (17)	0.86194 (18)	0.0292 (5)	
H14A	0.2765	1.2837	0.8809	0.035*	
C15	0.0967 (2)	1.24157 (19)	0.83757 (19)	0.0342 (6)	
H15A	0.0619	1.3121	0.8403	0.041*	
C16	0.0208 (2)	1.1563 (2)	0.80940 (19)	0.0357 (6)	
H16A	−0.0665	1.1670	0.7928	0.043*	
C17	0.0743 (2)	1.05449 (18)	0.80576 (18)	0.0272 (5)	

### Atomic displacement parameters (Å^2^)

**Table:**

	*U*^11^	*U*^22^	*U*^33^	*U*^12^	*U*^13^	*U*^23^
F1	0.0207 (6)	0.0479 (9)	0.0792 (12)	0.0004 (6)	−0.0048 (8)	−0.0278 (9)
O1	0.0206 (7)	0.0158 (6)	0.0451 (9)	0.0020 (5)	0.0062 (7)	−0.0057 (7)
O2	0.0263 (8)	0.0254 (7)	0.0522 (10)	0.0054 (6)	0.0151 (8)	−0.0038 (8)
O3	0.0184 (7)	0.0204 (7)	0.0501 (10)	−0.0006 (5)	0.0024 (7)	0.0084 (7)
O4	0.0185 (7)	0.0237 (7)	0.0259 (7)	−0.0023 (6)	−0.0066 (6)	0.0078 (6)
N1	0.0188 (8)	0.0191 (7)	0.0193 (7)	0.0021 (6)	0.0023 (7)	0.0041 (7)
C1	0.0184 (9)	0.0200 (8)	0.0232 (9)	0.0035 (7)	−0.0021 (8)	−0.0037 (8)
C2	0.0177 (9)	0.0168 (8)	0.0185 (8)	0.0039 (7)	0.0000 (7)	−0.0016 (7)
C3	0.0272 (11)	0.0329 (11)	0.0214 (10)	0.0072 (9)	−0.0055 (9)	−0.0034 (9)
C4A	0.0359 (14)	0.0264 (12)	0.0189 (11)	0.0043 (10)	−0.0072 (10)	0.0021 (10)
C4B	0.094 (16)	0.047 (13)	0.034 (12)	0.037 (13)	0.001 (12)	0.011 (11)
C5	0.0379 (13)	0.0284 (11)	0.0233 (10)	0.0024 (10)	0.0057 (10)	0.0093 (9)
C6	0.0192 (9)	0.0134 (8)	0.0310 (11)	0.0031 (7)	0.0015 (8)	0.0030 (8)
C7	0.0222 (10)	0.0232 (9)	0.0288 (11)	0.0031 (8)	−0.0111 (9)	−0.0023 (9)
C8	0.0569 (17)	0.0303 (12)	0.0485 (16)	0.0053 (12)	−0.0199 (14)	−0.0135 (12)
C9	0.0251 (12)	0.0356 (12)	0.0506 (16)	0.0086 (10)	−0.0058 (11)	0.0071 (12)
C10	0.0347 (13)	0.0404 (13)	0.0258 (11)	0.0022 (11)	−0.0086 (10)	0.0069 (10)
C11	0.0175 (9)	0.0159 (8)	0.0226 (10)	0.0030 (7)	0.0039 (7)	0.0000 (8)
C12	0.0175 (9)	0.0189 (8)	0.0180 (9)	0.0044 (7)	0.0027 (7)	−0.0007 (8)
C13	0.0195 (9)	0.0205 (9)	0.0236 (10)	0.0018 (7)	0.0026 (8)	−0.0019 (8)
C14	0.0350 (12)	0.0199 (9)	0.0329 (12)	0.0015 (9)	0.0066 (10)	−0.0026 (9)
C15	0.0430 (14)	0.0242 (10)	0.0354 (12)	0.0173 (10)	0.0045 (11)	0.0014 (10)
C16	0.0260 (12)	0.0402 (13)	0.0407 (13)	0.0178 (10)	−0.0068 (10)	−0.0068 (11)
C17	0.0184 (10)	0.0304 (11)	0.0326 (12)	0.0034 (8)	−0.0004 (9)	−0.0091 (10)

### Geometric parameters (Å, º)

**Table:**

F1—C17	1.356 (3)	C5—H5C	0.9900
O1—C1	1.325 (2)	C5—H5D	0.9900
O1—H1	0.89 (3)	C7—C9	1.513 (3)
O2—C1	1.201 (2)	C7—C8	1.517 (3)
O3—C6	1.228 (2)	C7—C10	1.518 (3)
O4—C6	1.337 (2)	C8—H8A	0.9800
O4—C7	1.483 (2)	C8—H8B	0.9800
N1—C6	1.348 (3)	C8—H8C	0.9800
N1—C5	1.465 (3)	C9—H9A	0.9800
N1—C2	1.474 (3)	C9—H9B	0.9800
C1—C2	1.536 (3)	C9—H9C	0.9800
C2—C11	1.540 (3)	C10—H10A	0.9800
C2—C3	1.557 (3)	C10—H10B	0.9800
C3—C4A	1.501 (3)	C10—H10C	0.9800
C3—C4B	1.502 (4)	C11—C12	1.513 (3)
C3—H3A	0.9900	C11—H11A	0.9900
C3—H3B	0.9900	C11—H7B	0.9900
C3—H3C	0.9900	C12—C17	1.391 (3)
C3—H3D	0.9900	C12—C13	1.389 (3)
C4A—C5	1.541 (4)	C13—C14	1.386 (3)
C4A—H4A	0.9900	C13—H13	0.9500
C4A—H4B	0.9900	C14—C15	1.388 (4)
C4B—C5	1.540 (4)	C14—H14A	0.9500
C4B—H4C	0.9900	C15—C16	1.376 (4)
C4B—H4D	0.9900	C15—H15A	0.9500
C5—H5A	0.9900	C16—C17	1.385 (3)
C5—H5B	0.9900	C16—H16A	0.9500
			
C1—O1—H1	108 (2)	N1—C5—H5D	111.2
C6—O4—C7	121.37 (16)	C4B—C5—H5D	111.2
C6—N1—C5	120.42 (17)	C4A—C5—H5D	73.1
C6—N1—C2	125.31 (16)	H5A—C5—H5D	134.9
C5—N1—C2	113.21 (17)	H5C—C5—H5D	109.1
O2—C1—O1	124.25 (19)	O3—C6—O4	124.63 (19)
O2—C1—C2	122.92 (19)	O3—C6—N1	123.33 (19)
O1—C1—C2	112.72 (16)	O4—C6—N1	112.04 (17)
N1—C2—C1	109.38 (15)	O4—C7—C9	110.42 (18)
N1—C2—C11	113.33 (16)	O4—C7—C8	109.64 (18)
C1—C2—C11	112.15 (16)	C9—C7—C8	112.8 (2)
N1—C2—C3	102.24 (16)	O4—C7—C10	101.69 (17)
C1—C2—C3	106.49 (16)	C9—C7—C10	110.92 (19)
C11—C2—C3	112.60 (15)	C8—C7—C10	110.8 (2)
C4A—C3—C2	105.08 (18)	C7—C8—H8A	109.5
C4B—C3—C2	106.1 (6)	C7—C8—H8B	109.5
C4A—C3—H3A	110.7	H8A—C8—H8B	109.5
C4B—C3—H3A	70.7	C7—C8—H8C	109.5
C2—C3—H3A	110.7	H8A—C8—H8C	109.5
C4A—C3—H3B	110.7	H8B—C8—H8C	109.5
C4B—C3—H3B	140.0	C7—C9—H9A	109.5
C2—C3—H3B	110.7	C7—C9—H9B	109.5
H3A—C3—H3B	108.8	H9A—C9—H9B	109.5
C4A—C3—H3C	141.3	C7—C9—H9C	109.5
C4B—C3—H3C	110.5	H9A—C9—H9C	109.5
C2—C3—H3C	110.5	H9B—C9—H9C	109.5
H3B—C3—H3C	70.3	C7—C10—H10A	109.5
C4A—C3—H3D	71.2	C7—C10—H10B	109.5
C4B—C3—H3D	110.5	H10A—C10—H10B	109.5
C2—C3—H3D	110.5	C7—C10—H10C	109.5
H3A—C3—H3D	136.3	H10A—C10—H10C	109.5
H3C—C3—H3D	108.7	H10B—C10—H10C	109.5
C3—C4A—C5	103.42 (19)	C12—C11—C2	114.72 (16)
C3—C4A—H4A	111.1	C12—C11—H11A	108.6
C5—C4A—H4A	111.1	C2—C11—H11A	108.6
C3—C4A—H4B	111.1	C12—C11—H7B	108.6
C5—C4A—H4B	111.1	C2—C11—H7B	108.6
H4A—C4A—H4B	109.0	H11A—C11—H7B	107.6
C3—C4B—C5	103.4 (2)	C17—C12—C13	116.22 (19)
C3—C4B—H4C	111.1	C17—C12—C11	121.26 (19)
C5—C4B—H4C	111.1	C13—C12—C11	122.51 (17)
C3—C4B—H4D	111.1	C14—C13—C12	121.8 (2)
C5—C4B—H4D	111.1	C14—C13—H13	119.1
H4C—C4B—H4D	109.0	C12—C13—H13	119.1
N1—C5—C4B	102.7 (6)	C15—C14—C13	119.9 (2)
N1—C5—C4A	101.83 (18)	C15—C14—H14A	120.1
N1—C5—H5A	111.4	C13—C14—H14A	120.1
C4B—C5—H5A	72.6	C16—C15—C14	120.1 (2)
C4A—C5—H5A	111.4	C16—C15—H15A	120.0
N1—C5—H5B	111.4	C14—C15—H15A	120.0
C4B—C5—H5B	141.5	C15—C16—C17	118.6 (2)
C4A—C5—H5B	111.4	C15—C16—H16A	120.7
H5A—C5—H5B	109.3	C17—C16—H16A	120.7
N1—C5—H5C	111.2	F1—C17—C16	118.07 (19)
C4B—C5—H5C	111.2	F1—C17—C12	118.55 (19)
C4A—C5—H5C	142.4	C16—C17—C12	123.4 (2)
H5B—C5—H5C	73.0		
			
C6—N1—C2—C1	−55.2 (2)	C3—C4A—C5—N1	−35.4 (2)
C5—N1—C2—C1	113.02 (19)	C3—C4A—C5—C4B	60.5 (8)
C6—N1—C2—C11	70.8 (2)	C7—O4—C6—O3	9.2 (3)
C5—N1—C2—C11	−121.03 (18)	C7—O4—C6—N1	−170.18 (16)
C6—N1—C2—C3	−167.78 (18)	C5—N1—C6—O3	2.7 (3)
C5—N1—C2—C3	0.4 (2)	C2—N1—C6—O3	170.11 (19)
O2—C1—C2—N1	−26.3 (3)	C5—N1—C6—O4	−177.96 (18)
O1—C1—C2—N1	157.34 (17)	C2—N1—C6—O4	−10.5 (3)
O2—C1—C2—C11	−152.9 (2)	C6—O4—C7—C9	−65.0 (2)
O1—C1—C2—C11	30.7 (2)	C6—O4—C7—C8	59.9 (3)
O2—C1—C2—C3	83.5 (2)	C6—O4—C7—C10	177.21 (18)
O1—C1—C2—C3	−92.9 (2)	N1—C2—C11—C12	60.3 (2)
N1—C2—C3—C4A	−23.3 (2)	C1—C2—C11—C12	−175.22 (17)
C1—C2—C3—C4A	−138.06 (18)	C3—C2—C11—C12	−55.1 (2)
C11—C2—C3—C4A	98.6 (2)	C2—C11—C12—C17	103.8 (2)
N1—C2—C3—C4B	21.3 (9)	C2—C11—C12—C13	−76.9 (2)
C1—C2—C3—C4B	−93.5 (9)	C17—C12—C13—C14	−0.7 (3)
C11—C2—C3—C4B	143.2 (9)	C11—C12—C13—C14	180.0 (2)
C4B—C3—C4A—C5	−60.8 (8)	C12—C13—C14—C15	0.9 (3)
C2—C3—C4A—C5	36.8 (2)	C13—C14—C15—C16	−0.5 (4)
C4A—C3—C4B—C5	60.9 (8)	C14—C15—C16—C17	−0.2 (4)
C2—C3—C4B—C5	−34.1 (16)	C15—C16—C17—F1	−179.1 (2)
C6—N1—C5—C4B	147.8 (9)	C15—C16—C17—C12	0.5 (4)
C2—N1—C5—C4B	−21.1 (9)	C13—C12—C17—F1	179.50 (19)
C6—N1—C5—C4A	−169.45 (18)	C11—C12—C17—F1	−1.2 (3)
C2—N1—C5—C4A	21.7 (2)	C13—C12—C17—C16	0.0 (3)
C3—C4B—C5—N1	33.3 (16)	C11—C12—C17—C16	179.3 (2)
C3—C4B—C5—C4A	−60.4 (8)		

### Hydrogen-bond geometry (Å, º)

**Table:**

*D*—H···*A*	*D*—H	H···*A*	*D*···*A*	*D*—H···*A*
C15—H15*A*···F1^i^	0.95	2.59	3.378 (3)	141
C16—H16*A*···O1^i^	0.95	2.60	3.541 (3)	173
O1—H1···O3^ii^	0.89 (3)	1.73 (3)	2.611 (2)	173 (3)

Symmetry codes: (i) −*x*, *y*+1/2, −*z*+3/2; (ii) −*x*+1, *y*−1/2, −*z*+3/2.

## References

[bb1] Anderson, J. C., Wu, N., Santoro, S. W., Lakshman, V., King, D. S. & Schultz, P. G. (2004). *Proc. Natl Acad. Sci. USA*, **101**, 7566–7571. 10.1073/pnas.0401517101 PMC419646 15138302

[bb2] Bernstein, J., Davis, R. E., Shimoni, L. & Chang, N.-L. (1995). *Angew. Chem. Int. Ed. Engl.* **34**, 1555–1573.

[bb3] Bruker (2009). *APEX2*, *SAINT* and *SADABS* Bruker AXS Inc., Madison, Wisconsin, USA.

[bb4] Cremer, D. & Pople, J. A. (1975). *J. Am. Chem. Soc.* **97**, 1354–1358.

[bb5] Jeng, A. Y., Savage, P., Beil, M. E., Bruseo, C. W., Hoyer, D., Fink, C. A. & Trapani, A. J. (2002). *Clin. Sci.* **103**, 98–101. 10.1042/CS103S102S 12193065

[bb6] Ryder, T. R., Hu, L. Y., Rafferty, M. F., Lotarski, S. M., Rock, D. M., Stoehr, S. J. & Szoke, B. G. (2000). *Drug Des. Discov.* **16**, 317–322. 10807036

[bb7] Sheldrick, G. M. (2008). *Acta Cryst.* A**64**, 112–122. 10.1107/S0108767307043930 18156677

[bb8] Spek, A. L. (2009). *Acta Cryst.* D**65**, 148–155. 10.1107/S090744490804362X PMC2631630 19171970

[bb9] Tamazyan, R., Karapetyan, H., Martirisyan, A., Martirosyan, V., Harutyunyan, G. & Gasparyan, S. (2004). *Acta Cryst.* C**60**, o390–o392. 10.1107/S0108270104007930 15178859

[bb10] Taylor, P. P., Pantaleone, D. P., Senkpeil, R. F. & Fotheringham, I. G. (1998). *Trends Biotechnol.* **16**, 412–418. 10.1016/s0167-7799(98)01240-2 9807838

